# Ligand-binding properties of XaffOBP9, a Minus-C odorant-binding protein from *Xyleborus affinis* (Coleoptera: Curculionidae: Scolytinae)

**DOI:** 10.3389/fphys.2023.1326099

**Published:** 2024-01-03

**Authors:** Qian Wang, Xiang Zhou, Kai Zhang, Lei Qin, Qi Wu, Linan Deng, Zheyuan Xu, Jixing Guo

**Affiliations:** Key Laboratory of Green Prevention and Control of Tropical Plant Diseases and Pests (Ministry of Education), School of Tropical Agriculture and Forestry, Hainan University, Haikou, China

**Keywords:** *Xyleborus affinis*, odorant-binding proteins, competitive binding assay, site-directed mutagenesis, key binding site

## Abstract

*Xyleborus affinis,* one of the most important pests of rubber trees, has caused severe damage to the natural rubber industry in Hainan province. The ability to detect host plants through a sensitive and specific olfactory system is crucial for *Xyleborus affinis*. Odorant binding proteins (OBPs) are believed to bind and carry hydrophobic active compounds from the environment to the surface of olfactory receptor neurons. To investigate the potential functional role of the highly expressed XaffOBP9 in binding with semiochemicals, we cloned and analyzed the cDNA sequence of XaffOBP9. The results showed that XaffOBP9 contains a 411bp open reading frame that encodes 136 amino acids. Then XaffOBP9 was expressed in *Escherichia coli.* The binding affinity of the recombinant OBP to 15 different ligands (14 host plant volatiles and 1 aggregation pheromone) was then examined using a fluorescence competitive binding approach. The results demonstrated that XaffOBP9 exhibited broad binding capabilities and strong affinities for 14 ligands. The structure of XaffOBP9 and its interactions with fourteen ligands were further analyzed by modeling and molecular docking, respectively. Based on the docking result, we found hydrophobic interactions are important between XaffOBP9 to these ligands and three amino acid residues (L71, Y106, and L114) were highly overlapped and contributed to the interaction with ligands. Mutation functional assays confirmed that the mutant L114A showed significantly reduced binding capacity to these ligands. This study suggested that XaffOBP9 may be involved in the chemoreception of semiochemicals and that it is helpful for the integrated management of *X. affinis*.

## Introduction

The rubber tree, *Hevea brasiliensis*, is the most important plant cultivated for rubber production (Guo et al., 2006; Cui et al., 2023) *X. affinis* (Coleoptera: Curculionidae: Scolytinae), one of the dominant ambrosia beetle species of rubber trees in Hainan province, has caused serious damage to the natural rubber industry. It primarily attacks unhealthy, damaged or felled trees, but it can also infest healthy stands (Merkl and Tusnádi, 1992). This pest mainly targets trunks and larger branches, rather than roots near the ground, and it can attack both slender and stout sections (Sobel et al., 2015). Additionally, *Xyleborus affinis* has known to attack over two hundred economically important plant species, including *Theobroma cacao*, *Mangifera indica* and *Saccharum officinarum* (Biedermann, 2020). This beetle has spread to tropical and subtropical regions worldwide, and international timber commerce facilitated its extension (Rabaglia et al., 2009; Gohli et al., 2016; Ospina-Garcés et al., 2021). Understanding the chemical ecology and host-seeking behavior of *X. affinis* is crucial for developing detection and control strategies. The primary method for monitoring ambrosia beetle populations involves the use of lures to attract host-seeking adults. While most species in the *Xyleborus* genus respond to ethanol, an indicator of stressed or dying trees and commonly used as an attractant lure, *X. affinis* is only weakly attracted to ethanol (Ranger et al., 2011; Steininger et al., 2015; Lehenberger et al., 2020; Cavaletto et al., 2021). Previous study has shown that plant volatiles can induce the olfactory response of *X. affinis*, with lauraceous host-based volatiles having a significant effect on attracting this beetle. The monoterpene α-pinene has been found to enhance the attractive effect of ethanol in *X. affinis* based on trapping investigations (Miller and Rabaglia, 2009). Electroantennogram (EAG) responses of female *X. affinis* to host plant volatiles have also been recorded, with essential oil extract from *Leptospermum scoparium* and *Phoebe porosa*, as well as silkbay wood of *P. humilis,* eliciting EAG responses in *X. affinis* (Hanula and Sullivan, 2008). Another study compared the EAG responses of laboratory-reared and wild *X. affinis* to host kairomones of *Bursera simaruba*, *Mangifera indica*, and *Persea schiedeana*, with the highest responses being triggered by *Bursera simaruba* at 48 h in *X. affinis* (Romero et al., 2022). The volatile profiles of aged bark samples, including compounds such as (-)-β-pinene, sabinene, ɑ-pinene, myrcene, camphene, 3-carene, m-cymene, (S)-(-)-limonene, ɑ-copaene, and terpinolene, varied. In addition, the aggregation pheromone, (s)-cis-verbenol, also exhibits attraction effects on this rubber bark beetle (Brand et al., 1975; Chen et al., 2019). (+)-longifolene, tetradecane, and 2-phenyl-2-propanol have shown an attraction effect on *X. affinis* (Xu et al., 2020). Insect behavior is primarily guided by complex olfactory cues (Sachse and Krieger, 2011; Renou and Anton, 2020). Insects have the ability to detect molecular information via odors in their environment and respond accordingly. Insect olfaction has evolved into a highly specific and sensitive chemical sensing system, involving a sophisticated chain reaction (Hansson and Stensmyr, 2011; Andersson, 2012). In the sensing process, odorant binding proteins (OBPs), a type of small, water-soluble proteins, play a crucial role in carrying hydrophobic odorants and pheromones from the external environment to the membrane of chemosensory neurons across the aqueous lymph of chemosensory (Pelosi et al., 2014; Venthur and Zhou, 2018; Rihani et al., 2021). These OBPs are characterized by interlocking disulfide bonds formed by conserved cysteines. According to the number of conserved cysteine residues, insect OBPs are categorized into four distinct types. These types are “Classic OBPs” (with six conserved cysteines), “Minus-C OBPs” (with four conserved cysteines), “Plus-C OBPs” (with eight conserved cysteines), and “Atypical OBPs” (with more than eight conserved cysteines) (Ha and Smith, 2022; Zafar et al., 2022). The connectivity of disulfide bridges creates an interior hydrophobic pocket for binding lipophilic ligands (Pelosi et al., 2014; Zhang et al., 2020). Compared to classical OBPs, research on minus-C OBPs, both in terms of molecular identification and functional analysis, is limited (Lagarde et al., 2011). Minus-C OBP is characterized by the presence of two disulfide bonds formed by four conserved cysteines. AmelOBP14 of *Apis mellifera* is the first identified minus-C OBP for which a 3D structure has been reported (Forêt and Maleszka, 2006; Spinelli et al., 2012). It has been suggested that minus-C OBPs may be ancestral proteins, and the loss of one disulfide bridge could potentially have functional relevance, as it could lead to the formation of a more flexible structure (Vieira and Rozas, 2011). To enhance our understanding of the molecular basis of host-seeking behavior in *X. affinis,* we have identified 10 candidate OBP genes by searching the transcriptome database of *X. affinis* adults. Among these, *XaffOBP9* was found to be highly expressed and may have a possible functional role in the host-seeking process. We have cloned and analyzed the cDNA sequence of XaffOBP9 and explored the ligand-binding mechanism based on the binding experiments and site-directed mutagenesis (Bachman, 2013). This study aims to provide evidence that XaffOBP9 is involved in the chemoreception of semiochemicals, with potential implications for the integrated treatment of *X. affinis*.

## Materials and methods

### Insect sample collection

Adults of *X. affinis* were obtained from the rubber forest located on the Danzhou Campus of Hainan University (19.51°N, 109.49°E). The entire bodies of the specimens were promptly frozen using liquid nitrogen and subsequently preserved at a temperature of −80°C until the isolation of RNA.

### Relative expression analysis of *XaffOBPs*

The RNAprep Pure Micro Kit (Tiangen, Beijing, China) was utilized to extract total RNA in accordance with the manufacturer’s instructions. The PrimeScript RT Reagent Kit with gDNA Eraser (Takara, Liaoning, China) was employed to synthesize cDNA and eliminate any potential genomic DNA contamination. The Real-time quantitative PCR (RT-qPCR) primers for the *XaffOBPs* genes were designed using Primer Premier v5.0 (Premier Biosoft, CA, United States) and are provided in Supplementary Table S1. β-actin was employed as an internal reference. The RT-qPCR was performed in three duplicates using the Applied Biosystems QuantStudio 5 real-time PCR system (Thermo Fisher Scientific, MA, United States) with ChamQ Universal SYBR qPCR Master Mix (Vazyme, Nanjing, China). The reaction conditions were 95°C for 30 s followed by 30 cycles of 95°C for 5 s, and 60°C for 30 s. The melting curve was analyzed to verify the amplification of a single fragment, and the relative expression was determined using the 2^−ΔΔCt^ method (Livak and Schmittgen, 2001). The significant difference of the expression level of *XaffOBP* genes were calculated using one-way analysis of variance with *p* < 0.05 by SPSS Statistics 18 (SPSS Inc., Chicago, United States).

### Sequence and phylogenetic tree analysis of XaffOBP9

The XaffOBP9 cDNA sequence and its corresponding amino acid sequence were analyzed using DNAMAN (Lynnon Biosoft, CA, United States). The N-terminal signal peptide sequences were predicted using SignalP V5.0 (https://services.healthtech.dtu.dk/services/SignalP-5.0/). The online program tools ProtParam (https://web.expasy.org/protparam/), SOPMA (https://npsa-prabi.ibcp.fr/cgi-bin), and ESPript3.0 (https://espript.ibcp.fr/ESPript/ESPript/index.php) software were used to predict the chemical and physical properties, secondary structure, and hydrophobicity scales of XaffOBP9. Multiple alignments were conducted using ClustalW (https://www.genome.jp/tools-bin/clustalw). The MEGA 5.2 program was used to construct the phylogenetic trees of XaffOBP9 with similar OBPs from other insect species by using the neighbor-joining method and a model that included the number of differences and the pairwise deletion of gaps.

### Expression and purification of XaffOBP9

Specific primers were designed to clone the cDNA that encodes XaffOBP9 (Supplementary Table S1). The PCR products were inserted into the pET-28a (+) vector using NotI and NcoI restriction endonucleases. The plasmid containing the correct insert fragment was subsequently transformed into *Escherichia coli* BL21 (DE3) cells. The recombinant protein was induced at 28°C for 6 h by 1 mM isopropyl β-d-l-thiogalactopyranoside (IPTG) when the OD600 value reached 0.6. The suspension was sonicated and then separated into supernatant and sediment by centrifugation (11,000 rpm, 20 min, 4 C). The protein was then purified using Ni-NTA 6FF (Sangon Biotech, Shanghai, China) in a graded imidazole series of 0 mM, 20 mM, 40 mM, 60 mM, 80 mM, 100 mM, 200 mM, 400 mM, 600 mM for washing and desalted using Dialysis Membrane (Sangon Biotech, Shanghai, China). The molecular weight and purity of the XaffOBP9 proteins were checked using 15% SDS-PAGE.

### Fluorescence competition binding assays of XaffOBP9

N-phentl-1-naphthylamine (1-NPN) was used and dissolved in chromatographic methanol (1 mM) as the fluorescent reporter. The purified XaffOBP9 was dissolved at a final concentration of 2 μM in 50 mM Tris-HCl (pH 7.4). The binding ability of the fluorescent probe to XaffOBP9 was measured using the Infinite 200 PRO microplate plate reader (Tecan, Mannedorf, Switzerland). Data for demonstrating the formation of [XaffOBP/1-NPN] complex were obtained by the titrating of 2 μM of protein with aliquots of 1-NPN to final concentrations ranging from 2 to 26 μM. Fluorescence of 1-NPN was excited at 337 nm, and the emission spectra were recorded between 370 and 550 nm at 25 C and 5-nm slits for emission with Costar^®^ 96-well microtiter plates (Corning, NY, United States).15 host volatiles related to the life activities of *X. affinis* were selected as candidate ligands (Table 1). These compounds (>90% puity) were purchased from Yuanye Bio-Technology (Shanghai, China), Alading Chemical Industry (Shanghai, China) and Sigma-Aldrich (MO, United States). Binding data were collected as three independent measurements. The IC50 values, which represent the concentrations of competitors that resulted in a 50% reduction in fluorescence intensity, were recorded and measured. The binding dissociation constants were calculated from the corresponding IC50 values using the formula: Ki = [IC50]/(1 + [1-NPN]/K_1-NPN_), where [1-NPN] is the free concentration of 1-NPN and *K*_*1-NPN*_ is the dissociation constant of the protein/1-NPN complex (Ban et al., 2003; Yang et al., 2022). The data analysis and binding curve calculations were processed in Prism 8 (Graphpad Software, San Diego, CA, United States).

**TABLE 1:** Binding affinities of the tested ligands to XaffOBP9.

No.	Ligands	Molecular formula	Plane structure	Purity (%)	CAS No.	IC50 (µM)	Ki (µM)
1	2-Phenyl-2-propanol	C_9_H_12_O	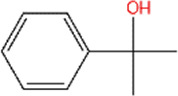	97%	617-94-7	14.0	12.0
2	Myrcene	C_10_H_16_	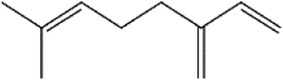	>90%	123-35-3	11.4	9.8
3	Methyl palmitate	C_17_H_34_O_2_	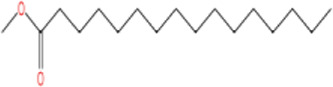	97%	112-39-0	-	-
4	(S)-cis-Verbenol	C_10_H_16_O	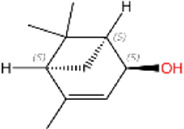	>95%	18,881-04-4	16.2	13.9
5	Camphene	C_10_H_16_	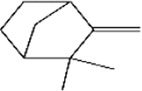	95%	79-92-5	9.3	8.0
6	α-pinene	C_10_H_16_	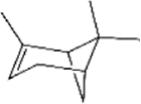	98%	80-56-8	9.9	8.5
7	3-Carene	C_10_H_16_	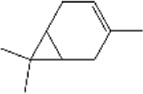	90%	13,466-78-9	12.4	10.6
8	α-copaene	C_15_H_24_	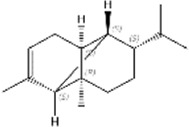	95%	3856-25-5	13.3	11.4
9	Terpinolene	C_10_H_16_	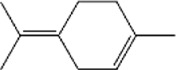	95%	586-62-9	13.2	11.3
10	(+)-Longifolene	C_15_H_24_	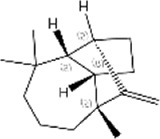	95%	475-20-7	19.5	16.7
11	Sabinene	C_10_H_16_	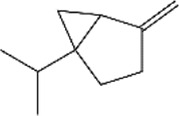	>98%	3387-41-5	18.2	15.6
12	(-)-β-pinene	C_10_H_16_	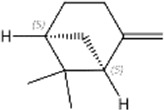	98%	18,172-67-3	16.0	13.7
13	Tetradecane	C_14_H_30_	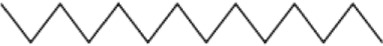	99%	629-59-4	15.7	13.5
14	m-Cymene	C_10_H_14_	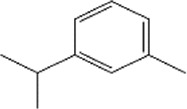	>99%	535-77-3	17.3	14.8
15	(S)-(-)-Limonene	C_10_H_16_	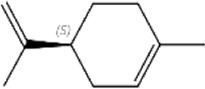	96%	5989-54-8	16.4	14.0

-, IC50 could not be calculated because no *Ki* value was detected in binding assay.

### Molecular modeling and docking

The model of XaffOBP9 was predicted using the I-TASSER (Zhou et al., 2022) (https://seq2fun.dcmb.med.umich.edu/I-TASSER/) online program. The 3D model was visualized using PYMOL Viewer (http://www.pymol.org) and Discovery Studio visualizer (BIOVIA, CA, United States). The 3D structures of the 15 ligands were downloaded from PubChem (https://pubchem.ncbi.nlm.nih.gov/). Molecular docking was conducted using Autodock (Molecular Graphics Laboratory, CA, United States) (Forli et al., 2016). The default parameters were used as described in the Autodock manual. The potential binding pockets of WT and three mutants were predicted using DoGSite3 (https://proteins.plus/) online program (Volkamer et al., 2010; Volkamer et al., 2012; Graef et al., 2023).

### Site-directed

Three types of XaffOBP9 mutants (L71A, L114A, Y106A) were generated using the Fast Site-Directed Mutagenesis Kit (Tiangen, Beijing, China) according to manufacturer protocol. Sited-directed mutagenesis primers were listed in Supplementary Table S1 The PCR amplification reaction was 95°C for 2 min, followed by 94 C for 20 s, 55 C for 10 s, 68 C 2.5 min, with final incubation at 68 °C for 5 min. The three mutant proteins were then expressed and purified following the procedures used for wild-type XaffOBP9.

## Results

### Identification and expression of OBP genes in *Xyleborus affinis*

A total of approximately 5.74 GB of clean data (Accession number: PRJNA1046655) was obtained from the RNA sample extracted from *X. affinis* adults. The accuracy of the sequencing was confirmed by Q30 percentages of 93.25% and a GC content of 41.31%, indicating the credibility of the data (Supplementary Table S2). The reads from the sequencing successfully assembled into 67,228 transcripts, with an average length of 2,684 bp and an N50 length of 4,781 bp. These transcripts were further clustered and assembled into 27,786 unigenes, with a mean length of 1,250 bp and an N50 of 2,761 bp.

**FIGURE 1:**
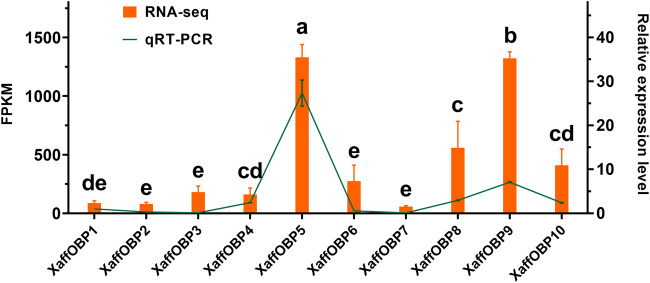
The expression profiles of XaffOBPs using RNA-seq and RT-qPCR. Different lowercase letters indicate the significant differences in the expression level of *XaffOBP*s genes measured by RT-qPCR.

Based on Nr annotation, ten unigenes were identified as OBP genes. All ten *XaffOBPs* were found to be full-length genes encoding complete open reading frames (ORFs), ranging in size from 133 to 228 amino acid residues. According to RT-qPCR results, the transcript levels of *XaffOBP9* and *XaffOBP5* were highly expressed in female adults, which were consistent with FPKM (Fragments Per Kilobase of exon model per Million mapped fragments) values obtained from RNA-Seq (Figure 1).

### Sequence analysis of XaffOBP9

**FIGURE 2:**
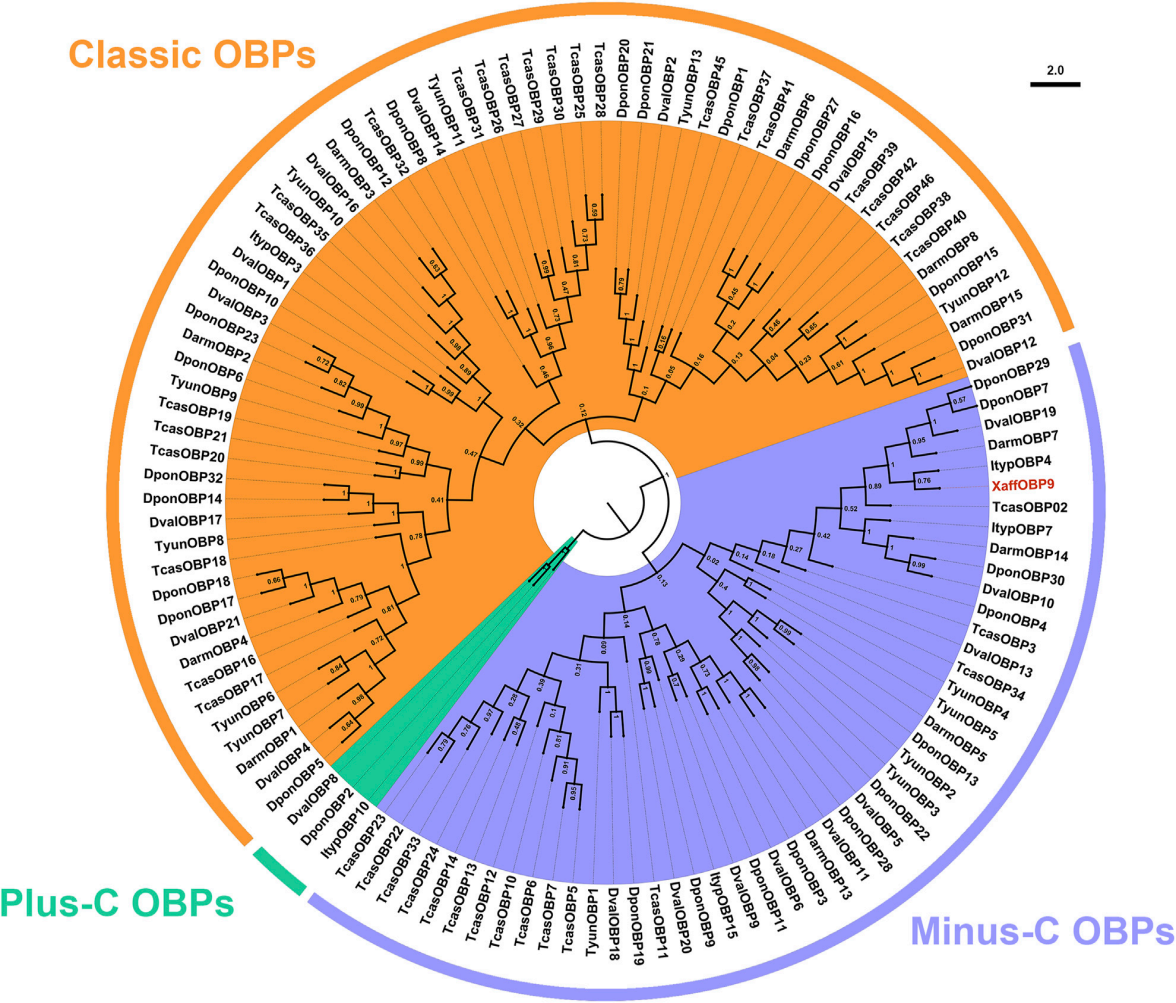
Phylogenetic tree of XaffOBP9 amino acid sequences with OBP from other species of Scolytinae. *Dendroctonus ponderosae* (Dpon), *Tribolium castaneum* (Tcas), *Dendroctonus armandi* (Darm), *Ips typographus* (Ityp), *Tomicus yunnanensis* (Tyun) and *Dendroctonus valens* (Dval). The tree was constructed using the neighbor-joining method with a bootstrap of 1,000 replicates. Thick arcs indicate phylogenetic subfamilies (Classic in orange, Minus-C in purple and Plus-C in green).

The cDNA sequence of XaffOBP9 was cloned and submitted to the NCBI GenBank database under the accession number OR499868. Sequence analysis revealed that the completed ORF of XaffOBP9 contained 411 nucleotides, encoding a protein of 136 amino acid residues. A 19-amino acid signal peptide was predicted using SignalP 5.0 (Supplementary Figure S1). The predicted molecular weight was 14.28 kDa, and the isoelectric point was 6.64 after the signal peptide was removed. BLASTx analysis showed that XaffOBP9 exhibited a maximum identity of 64.7% with DponOBP29 from *Dendroctonus ponderosae* (AGI05182.1), followed by OBPs from *Pagiophloeus tsushimanus* (64%, UWL63301.1), *Dendroctonus armandi* (61%, ALM64969.1), and *Pachyrhinus yasumatsui* (58%, WJJ63286.1). The sequence alignment of XaffOBP9 with five OBPs from other beetles revealed that XaffOBP9 possesses four cysteine residues and a conserved signature: X_33_-C_1_-X_30_-C_2_-X_39_-C_3_-X_16_-C_4_-X_12_ (X denotes any amino acid) (Supplementary Figure S2). Two disulfide bridges were observed between ɑ1 and ɑ3, ɑ5 and ɑ6. The phylogenetic tree was constructed using XaffOBP9, and OBP sequences from other beetles to evaluate the evolutionary relationships among proteins. The results showed that OBPs were divided into three groups: the Classic OBP clade with six conserved cysteines (76 OBPs), the Plus-C OBP group (4 OBPs), and the Minus-C subfamily, which included XaffOBP9 and other OBPs (Figure 2).

### Expression, purification, and binding assays of XaffOBP9

**FIGURE 3:**
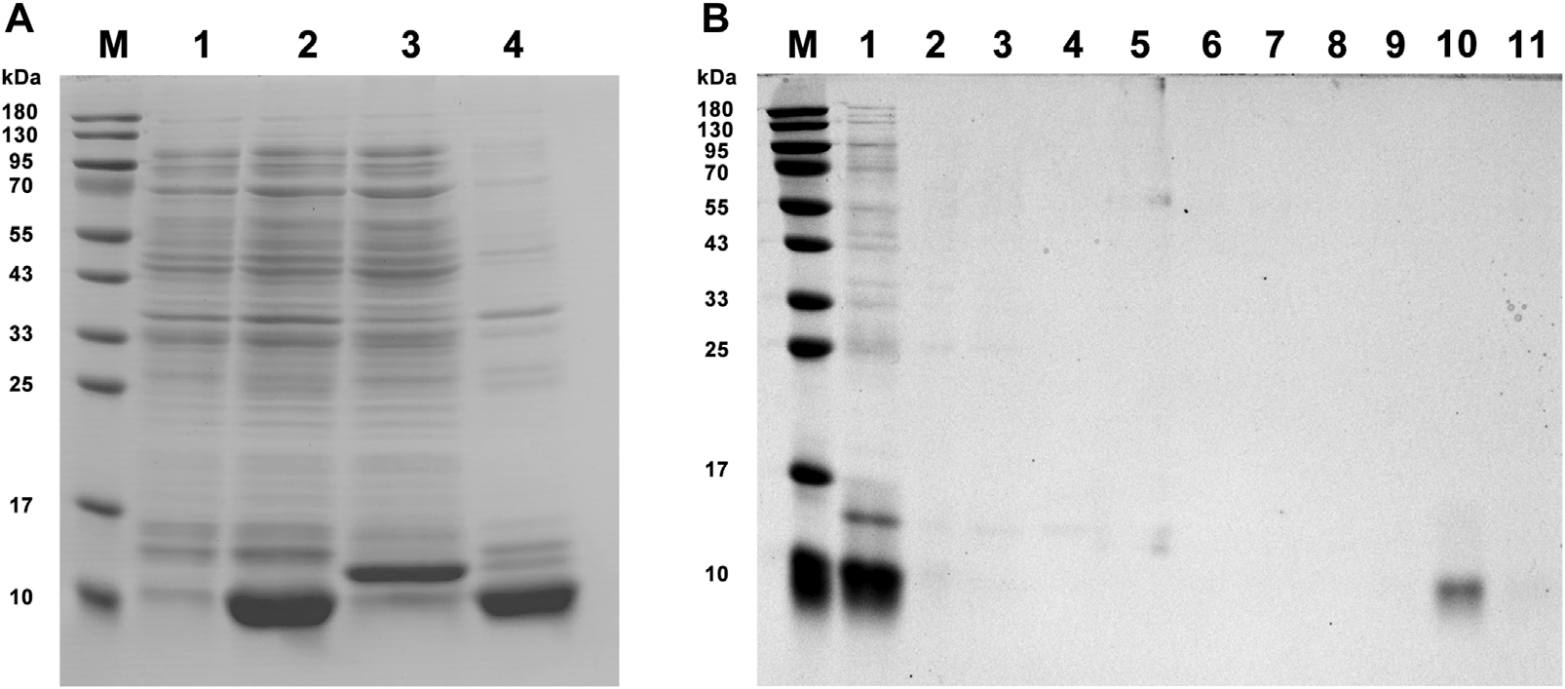
SDS-PAGE analysis of recombinant protein XaffOBP9 expression and purification. **(A)**: lane M, molecular weight marker of standard protein; lane 1, Non-induced BL21 bacteria within pET-28a/XaffOBP9 vector; lane 2, induced BL21 bacteria within pET-28a/XaffOBP9 vector; lane 3, supernatant of induced BL21 bacteria within pET-28a/XaffOBP9 vector; lane 4, the sediment of induced BL21 bacteria within pET-28a/XaffOBP9 vector; **(B)**: lane M, molecular weight marker of standard protein; lane 1, sediment of induced BL21 bacteria within pET-28a/XaffOBP9 vector; lane 2, protein fluid after flowing through the column; lane 3-11, samples were obtained using imidazole elution at different concentrations (0, 20, 40, 60, 80, 100, 200, 400 and 600 mM imidazole).

**FIGURE 4:**
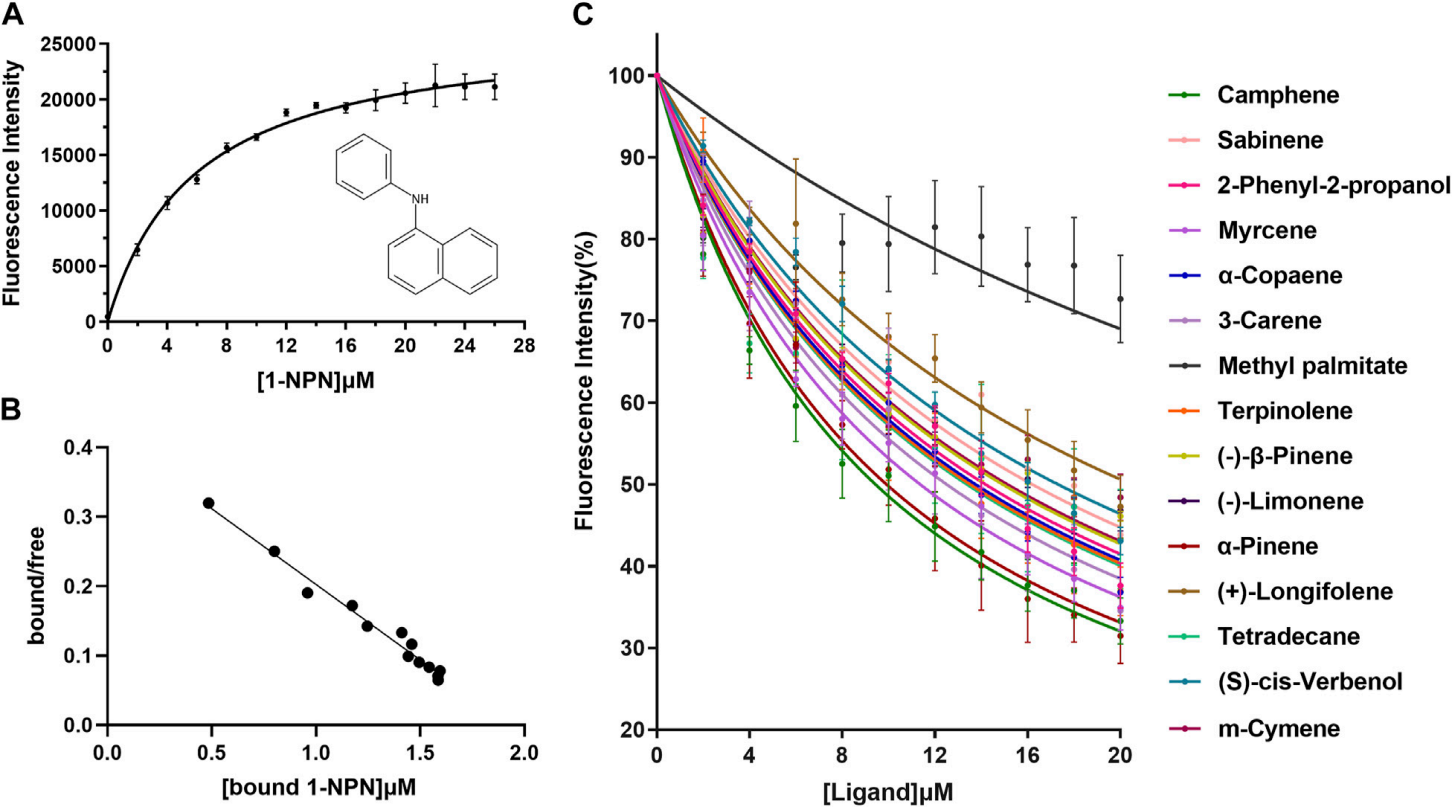
Fluorescence competitive binding assay. **(A)** and **(B)** Binding curves and Scathard plots of XaffOBP to the 1-NPN. **(C)** Competitive binding curves for XaffOBP9 with 15 test compounds. Data are the means of three independent duplicates.

Recombinant XaffOBP9 was successfully expressed in BL21 (DE3) *E. coli* cells as inclusion body form. SDS-PAGE analysis confirmed the presence of the expected band representing the induced XaffOBP9 with the predicted molecular mass (Figure 3A). The protein was then purified using Ni-IDA resin affinity chromatography (Figure 3B). The binding curve and Scatchard plots showed that XaffOBP9 exhibited a high affinity for the reporter 1-NPN, with a dissociation constant *K*_*1-NPN*_ of 5.91 µM (Figures 4A,B). The *K*_*1-NPN*_ value was used to calculate the *Ki* values of test ligands for XaffOBP9. The competitive fluorescence binding curves indicated that all test ligands reduced the relative fluorescence intensity of the [XaffOBP/1-NPN] mixture (Figure 4C). However, only methyl palmitates did not reduce the relative fluorescence intensity to less than half. For the other ligands, excluding methyl palmitate, they exhibited high binding affinity to XaffOBP9 with *Ki* values below 16 µM. Camphene and ɑ-pinene demonstrated the highest binding affinity to XaffOBP9, with *Ki* values of 8.0 and 8.5, respectively (Table 1).

### 3D modeling and molecular docking

The 3D structure revealed that the C-terminal region forms a wall over the binding pocket adjacent to the ɑ3 and ɑ6 (Supplementary Figure S3). The quality and accuracy of the predicted model were evaluated. The Ramachandran plot showed that 93.3% of the residues were in the most appropriate regions, which exceeds the criterion for judging rationality (90%). Additionally, 93.16% of the residues had an averaged 3D-one-dimensional score of >0.1 using Verify-3D, while the verification score of the final XaffOBP9 model using ERRAT was 100. Therefore, the predicted 3D model of XaffOBP9 is considered reasonable and reliable (Supplementary Figure S4).

**FIGURE 5:**
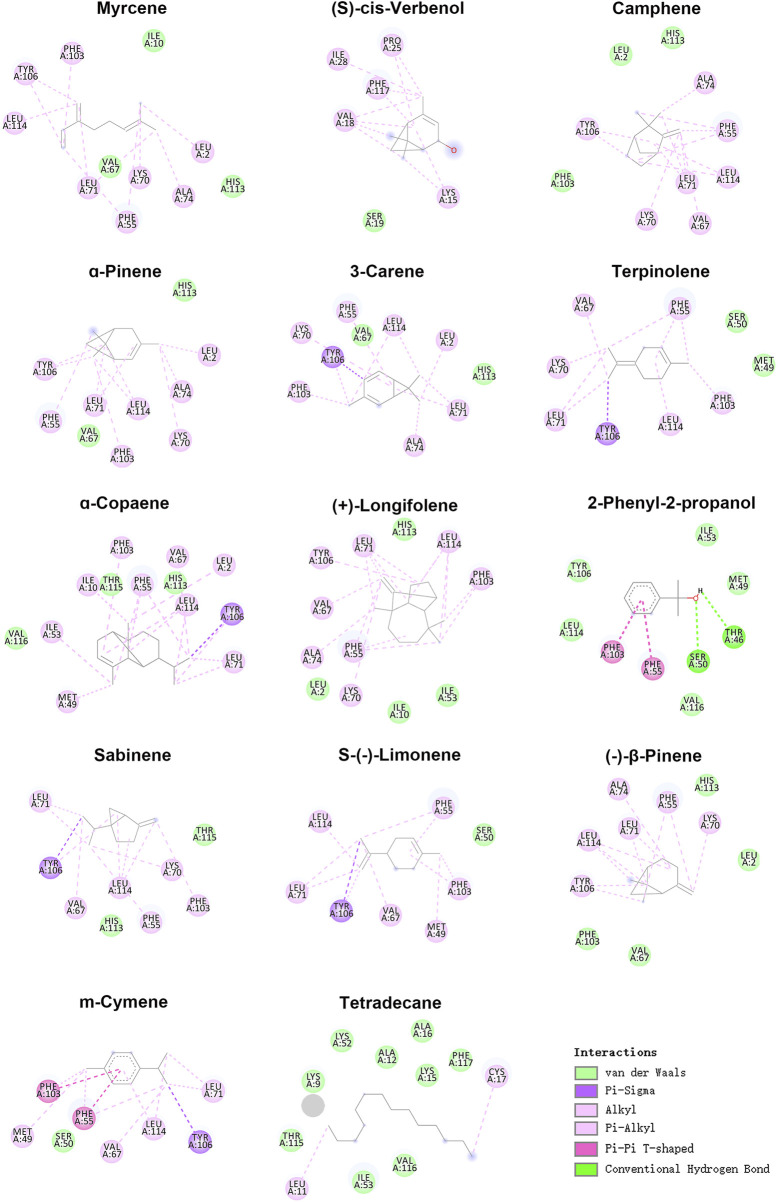
2D predicted interaction view of XaffOBP9 with 14 ligands. Pink and purple circles represented amino acid residues that had hydrophobic interactions with these ligands; dark green circles were amino acid residues that form hydrogen bonds with these ligands; light green circles were the amino acid residues with a van der Waals forces with these ligands.

**FIGURE 6:**
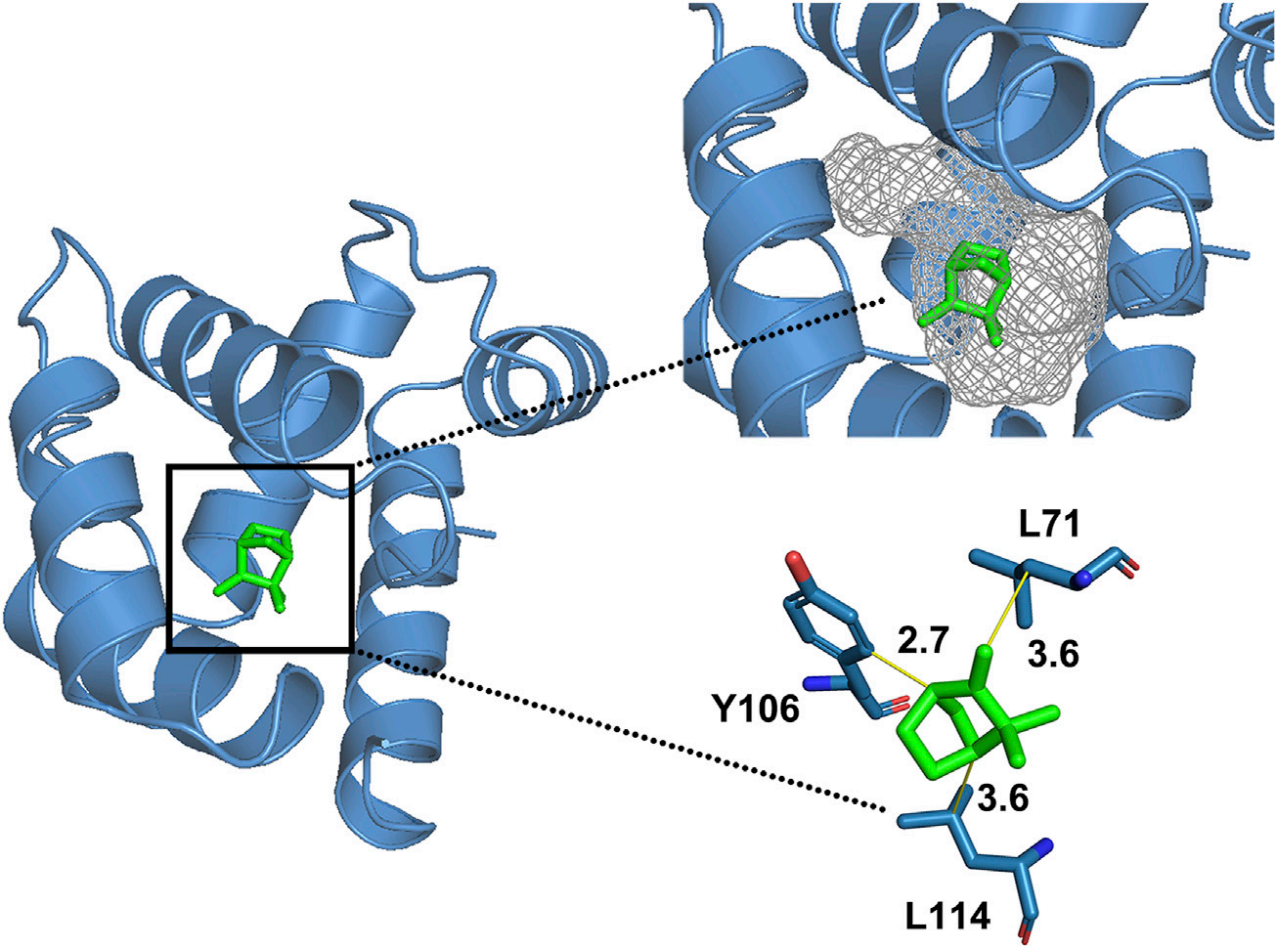
Molecular docking of XaffOBP9 with camphene. The binding pocket was marked by meshes.

We selected 14 compounds for docking analysis with the 3D model of XaffOBP9 to investigate the key binding sites of XaffOBP9 that engage with various ligands. Docking results demonstrated that XaffOBP9 binds to these 14 ligands, with free binding energies ranging from −3.0 kcal/mol to −6.9 kcal/mol (Table 2). Notably, camphene and terpinolene exhibited the strongest interaction with XaffOBP9, with binding energies of −6.9 kcal/mol. The main interaction between XaffOBP9 and its ligands primarily relies on Van der Waals and hydrophobic interactions, with amino acid residues such as His113, Val116, Ile10, and Ser50 participating in the formation of Van der Waals interactions and Leu2, Phe55, Lys70, Leu71, Ala74, Phe103, Tyr106, and Leu114 contributing to the formation of hydrophobic interactions (Figure 5). Only two amino acid residues, Tyr46 and Ser50, are involved in H-bond formation with 2-phenyl-2-propanol (Table 2). Three amino acid residues, L71, L114 and Y106 were highly overlapped and contributed to the interaction with ligands (Table 2). Take camphene, the ligand with the best binding affinity to XaffOBP9, as an example, it was located in the centre of a hydrophobic tunnel and interacted with L71 (3.6 Å), Y106 (2.7 Å), and L114 (3.6 Å) through alkyl interactions (Figure 6).

**TABLE 2:** Prediction of key amino acid residues during the docking of XaffOBP9 to different ligands.

No.	Ligand	Binding energy	Hydrophobic interactions	Van der Waals interactions	H-Bond
1	2-Phenyl-2-propanol	−6.5	Phe103 Phe55	Ile48 Met49 Ile53 **Tyr106 Leu114** Val116	Tyr46 Ser50
2	Myrcene	−4.0	Leu2 Phe55 Lys70 **Leu71** Ala74 Phe103 **Tyr106 Leu11**4	Ile10 Val67 His113	
3	Terpinolene	−6.9	Met 49 Phe55 Val67 Lys70 Lys71 **Leu71 Tyr106 Leu114** Ser50		
4	(S)-cis-Verbenol	−5.0	Lys15 Val18 Pro25 Ile28 Phe117	Ser19	
5	Camphene	−6.9	Phe55 Val67 Lys70 **Leu71** Ala74 **Tyr106 Leu114**	Leu2 Phe103	
				His113	
6	ɑ-Pinene	−6.7	Leu2 Phe55 Lys70 **Leu71** Ala74 Phe103 **Tyr106 Leu114**	Val67 His113	
7	3-Carene	−6.6	Leu2 Phe55 Lys70 **Leu71** Ala74 Phe103 **Tyr106 Leu114**	Val67 His113	
8	ɑ-Copaene	−6.8	Leu2 Ile10 Met49 Ile53 Phe55 Val67 **Leu71** Phe103 **Tyr106 Leu114**	His113 Thr115	
				Val116	
9	(+)-Longifolene	−5.5	Phe55 Val67 Lys70 **Leu71** Ala74 Phe103 **Tyr106** His113 **Leu114**	Leu2 Ile10	
				Ile53	
10	Sabinene	−6.5	Phe55 Val67 Lys70 **Leu71** Phe103 **Tyr106 Leu114**	His113 Thr115	
11	(-)-β-Pinene	−6.6	Phe55 Lys70 **Leu71** Ala74 **Tyr106 Leu114**	Phe103 Leu2 Val67 His113	
12	Tetradecane	−3.0	Leu11 Cys17	Lys9 Ala12 Lys15 Ala16 Lys52 Ile53 Thy115 Val116 Phe117	
13	m-Cymene	−6.7	Met49 Phe55 Val67 **Leu71** Phe103 **Tyr106 Leu114**	Ser50	
14	(S)- (-)-Limonene	−6.7	Met49 Phe55 Val67 **Leu71** Phe103 **Tyr106 Leu114**	Ser50	

Note: Key residues (overstriking) that bound to more than one ligand was selected as the object of subsequent site-directed mutation analysis.

### Site-directed mutagenesis of XaffOBP9

**FIGURE 7:**
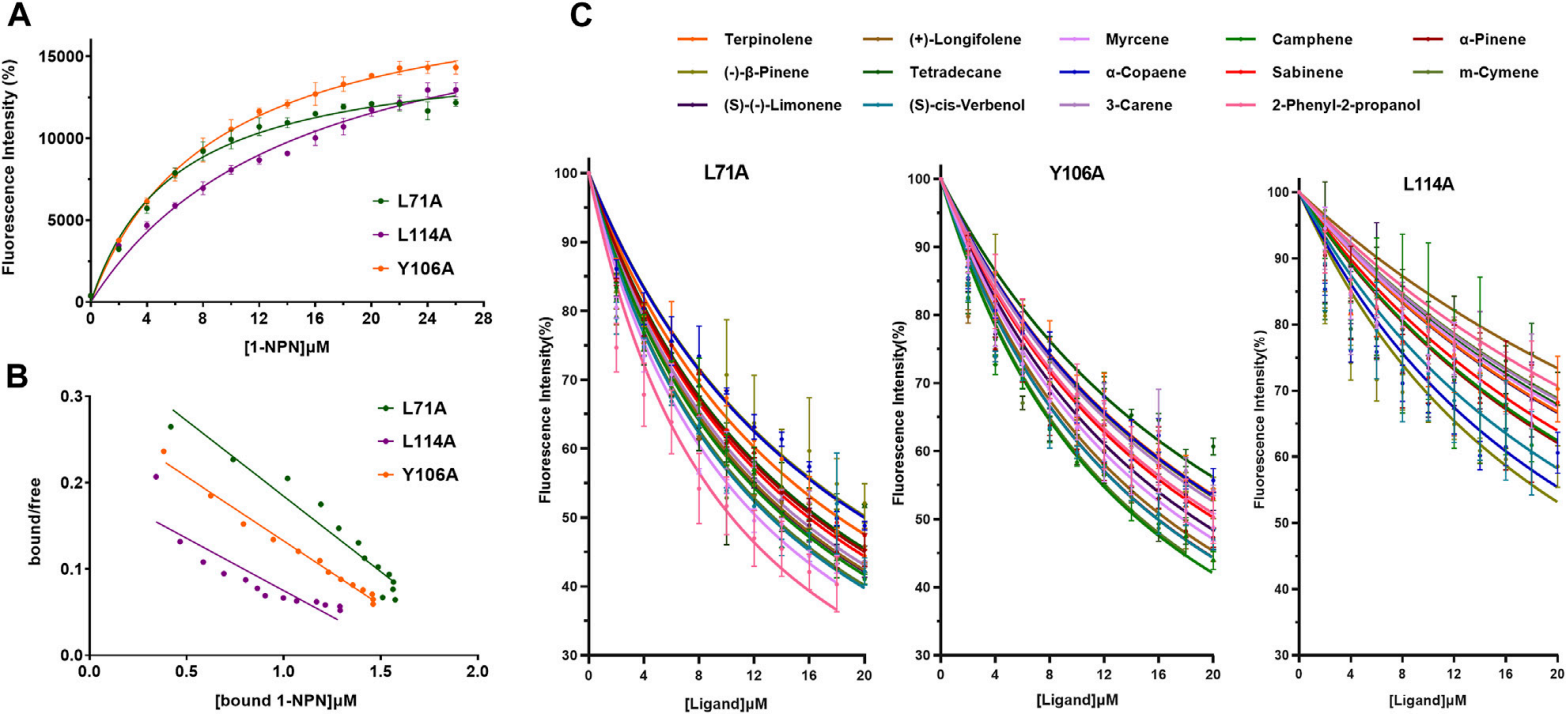
Competitive binding curves of 14 ligands to the mutant proteins. **(A)** Binding curves of L71A, Y106A and L114A with 1-NPN. **(B)** Scatchard plots of 71A, Y106A and L114A with 1-NPN. **(C)** Competitive binding curves of 14 ligands to three mutants.

**FIGURE 8:**
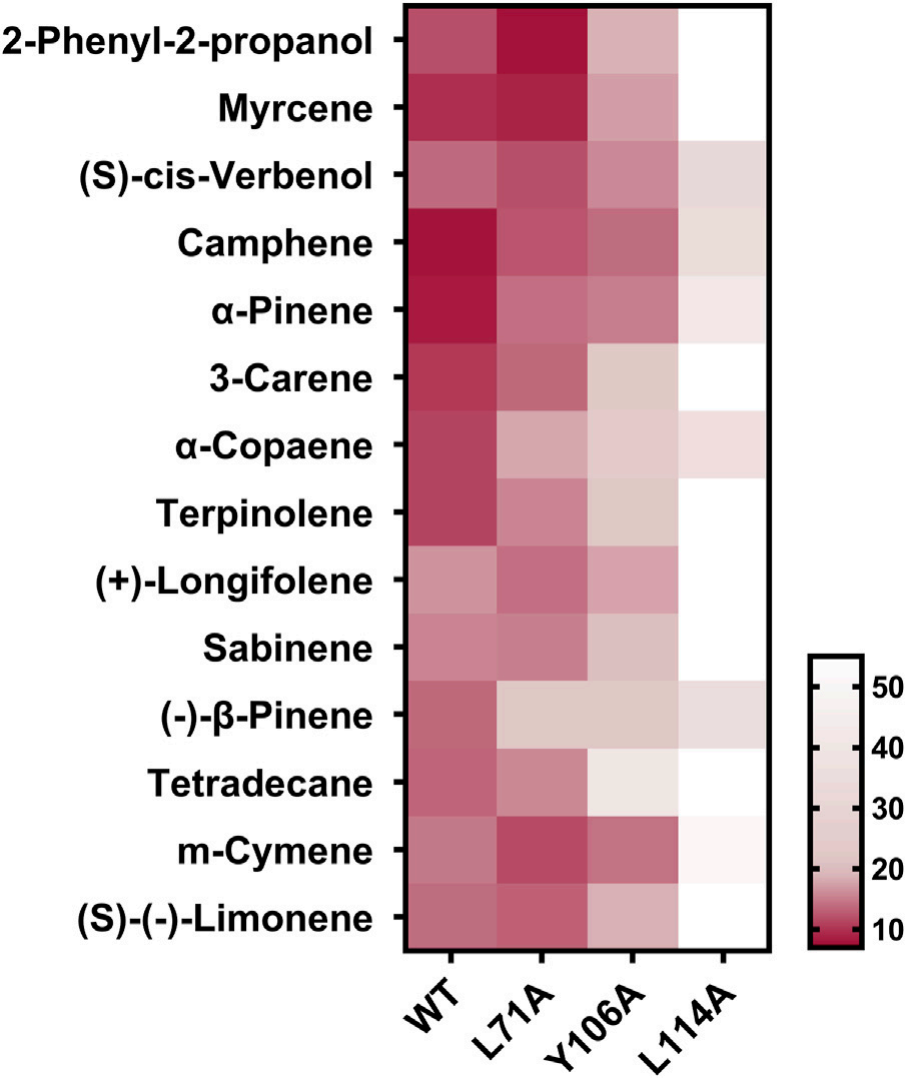
Heat map of the binding affinities (indicated by *Ki*) for WT (XaffOBP9) and three mutant proteins to 14 ligands. The tested ligands binding affinity with XaffOBP9 to be high if the *Ki* values < 10 μM, medium if 10 μM < *Ki* < 20 μM, weak if *Ki* > 20 μM and not bind if *Ki* > 50 μM. (Color depth is negatively correlated with *Ki* value). Color depth is negatively correlated with Ki value.

**FIGURE 9:**
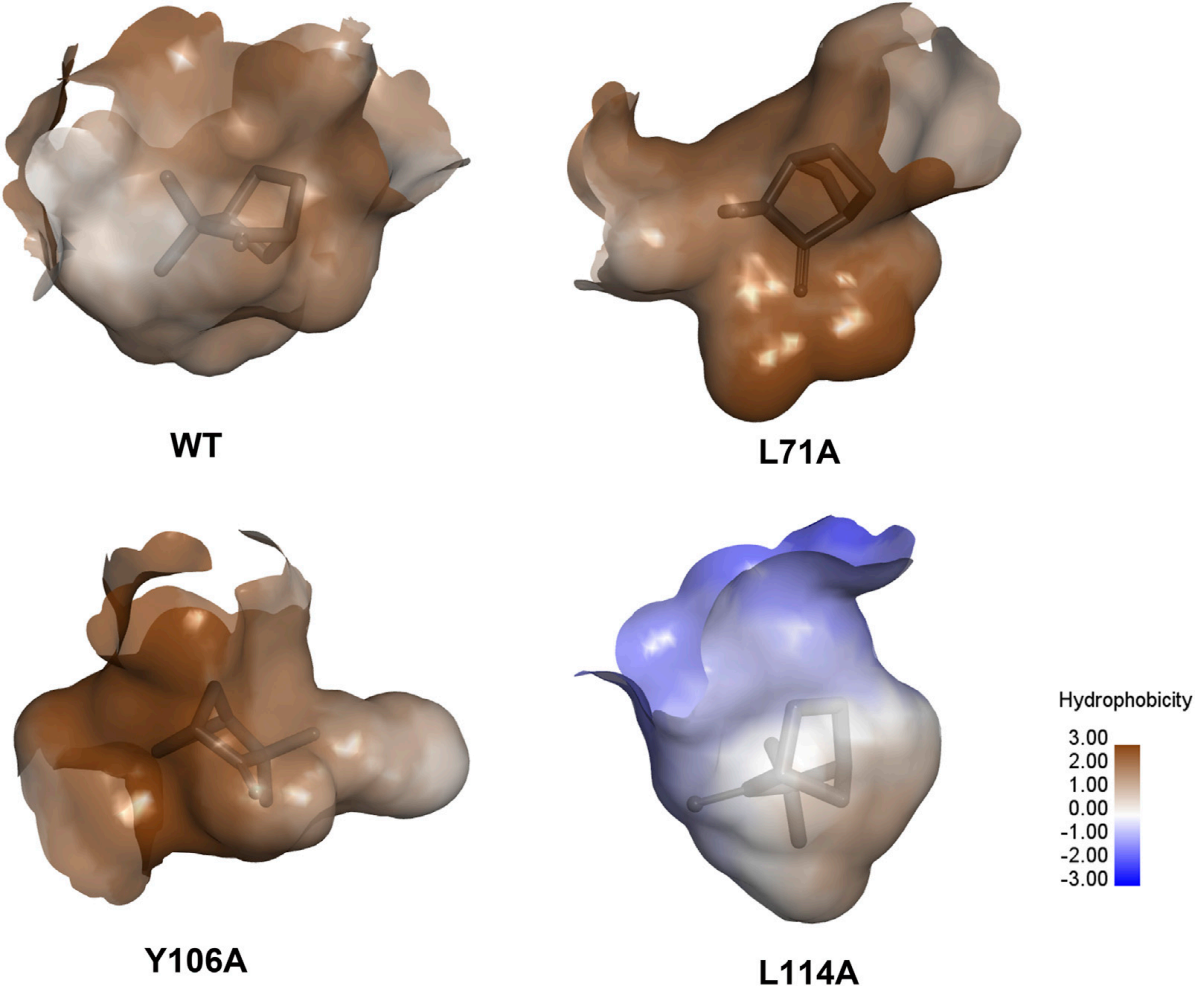
Prediction of protein-ligand binding pockets of WT and three mutant proteins.

To further clarify the binding mechanism of XaffOBP9 with ligands, key binding sites predicted by docking results were selected and mutated. Using site-directed mutagenesis, we replaced L71, Y106 and L114 with alanine, and generated three mutants L71A, Y106A and L114A. The mutant proteins were expressed and purified using the same procedures used as the wild-type XaffOBP9 (WT) (Supplementary Figure S5). The molecular mass of each protein corresponded to the predicted value. The *Kd* values of three mutant proteins, L71A, Y106A and L114A, with 1-NPN were 5.97 µM, 8.7 µM and 14.8 µM, respectively (Figures 7A,B). The results showed the interaction between XaffOBP9 and 1-NPN reporter had been affected by the mutations, with the mutant L114A demonstrating the most significant change. The binding ability of the 14 compounds with three mutant proteins was tested (Figure 7C). The L114A mutant showed the most significant loss in binding capacity to these ligands. The Y106A mutant exhibited a slight reduction in binding affinity for these ligands, but there was a noticeable decrease in its affinity for tetradecane. In contrast, almost no differences were found between the mutant L71A and WT (Figure 8). Additionally, the binding pockets for the three mutant proteins were predicted and assessed. The predicted binding pockets of all three mutant proteins were divided into three independent small pockets, while the docking results showed that the ligand was stably docked in one of the pockets. The appearance and size of the binding pockets in which the ligands docked had changed (Figure 9). The volume and depth of the L114A mutant had significantly decreased compared to the WT (Table 3). The pocket of L71A and Y106A was surrounded by hydrophobic residues, while the pocket of L114A consisted of polar residues on the top and hydrophobic residues on the bottom.

**TABLE 3:** Prediction of binding pockets of WT and three mutants.

Protein	Volume [Å^3^]	Surface [Å^2^]	Depth [Å]
WT	305.15	287.19	12.52
L71A	261.12	390.19	15.53
Y106A	167.42	187.31	12.42
L114A	77.31	201.72	6.65

## Discussion

Almost all insect behavior is related to olfactory perception (Sato and Touhara, 2009; Clark and Ray, 2016; Rihani et al., 2021). Insects rely on their sensitive olfactory system to detect plant volatiles and pheromones (Hansson and Stensmyr, 2011; Deisig et al., 2012), but the specific molecular mechanisms of olfaction in *X. affinis* for locating hosts have not been fully understood. Identifying the proteins responsible for olfactory perception is crucial to further comprehending the molecular mechanisms of the olfactory system (Clark and Ray, 2016; Zafar et al., 2022).

In this study, 10 OBP genes were identified from *X. affinis,* with *XaffOBP9* being highly expressed and potentially playing a functional role in olfactory perception. To investigate the molecular mechanism of XaffOBP9 in semiochemical detection, we cloned and analyzed the cDNA sequence of XaffOBP9. Multiple sequence alignment revealed that XaffOBP9 possesses four cysteine residues and a conserved signature, suggesting that it could belong to the Minus-C OBP subfamily.

Phylogenetic analysis also revealed that XaffOBP9 was grouped with Minus-C OBPs from other coleopteran insects within the Minus-C family branch. These findings suggest that these proteins are evolutionarily conserved and may have similar biological functions. Classic OBPs, characterized by six cysteine residues, form three disulfide bonds that are crucial in creating the binding pocket and stabilizing the three-dimensional structure (Zhang et al., 2011; Pelosi et al., 2014; Zafar et al., 2022). Previous studies suggested that the loss of one disulfide bridge in minus-C OBPs might have functional relevance, as it could generate a more flexible structure (Vieira and Rozas, 2011; Li et al., 2015; Zheng et al., 2016). AmelOBP14 structures in complex with 1-NPN, eugenol and citralva were investigated to explain their strong binding activity. By introducing a double cysteine mutant, in which the third disulfide bridge of classical OBPs was reintroduced. The addition of the new disulfide bridge caused constricted flexibility, affecting the ability to adapt its binding pocket to different odorants (Spinelli et al., 2012; Zhuang et al., 2014). BhorOBPm2, a Minus-C OBP in *Batocera horsfieldi* processes a larger binding pocket and broader ligand specificity (Zheng et al., 2016). The binding range of ligands and the intensity of binding with specific ligands were also influenced by the conformational flexibility of DhelOBP21 in *Dastarcus helophoroides* (Li et al., 2015). The binding assay results also demonstrated that XaffOBP9 exhibits broad binding capabilities. XaffOBP9 was able to bind to thirteen volatiles and one aggregation pheromone with high affinities, indicating its likely involvement in host volatiles recognition.

To further investigate the binding activities of XaffOBP9 to semiochemicals, molecular docking methods were implemented to estimate the free energy of ligand-receptor binding and analyze the conformations of ligands adopted within the binding sites of proteins. Docking results indicate that the main linkages between XaffOBP9 and the ligands were Van der Waals interactions and hydrophobic interactions. Moreover, the interactions and contributions of key amino acid residues of XaffOBP9 were characterized through the docking simulations. Numerous studies have demonstrated the hydrophobic interactions between insect OBPs and their ligands (Zhuang et al., 2014; Wen et al., 2019; Zhang et al., 2020). For instance, research on AlucOBP22 of *Apolygus lucorum* indicated that both β-ionone and β-caryophyllene were found within the pocket of AlucOBP22, in close proximity to hydrophobic amino acid residues. This suggests that hydrophobic interactions play a crucial role in ligand-specific binding (Liu et al., 2019). The presence of hydrophobic forces assists the protein in reducing its exposure to water, thereby maintaining its stable conformation and proper function (Zhuang et al., 2014; Wen et al., 2019). Our analysis of the binding models of XaffOBP9 to 14 ligands also revealed the involvement of hydrophobic interactions, indicating that its ligand binding mechanism is consistent with that of other insects.

It is noteworthy that three amino acid residues, L71, L114 and Y106 were highly overlapped and contributed to the interaction with ligands. These three amino acid residues were possibly involved in the binding with these ligands. Then we further explore the actual interaction by site-direct mutant. Three mutants of XaffOBP9 were obtained and analyzed. Subsequently, fluorescence binding assays revealed that the L114A mutant protein lost its ability to bind eight ligands, including 2-phenyl-2-propanol, myrcene, terpinolene, 3-carene, (+)-longifolene, sabinene, (s)- (-)-Limonene, and tetradecane, Furthermore, it weakly bound to six ligands, including camphene, (s)-cis-Verbenol, ɑ-pinene, ɑ-copaene, (-)-β-pinene, m-cymene. These findings suggest that the residue L114 may play a crucial role in the binding affinity of XaffOBP9 to distinct ligands. The mutant protein Y106A exhibited a certain decrease in binding to the ligands, especially to tetradecane and 1-NPN, suggesting the importance of Y106A as a binding site in XaffOBP9 due to its potential involvement in establishing specific hydrophobic interactions. Conversely, the L71A mutant protein showed only a slight decrease in its ability to bind these compounds, therefore we predicted that L71A may not be a critical binding site in XaffOBP9.

Proteins have pockets on the surface or inside that are suitable for ligand binding, and the amino acid residues around the pockets determine their shape, location, physical and chemical properties, and function (Stank et al., 2016). Therefore, we speculate that the location and shape of the binding pocket of WT may be altered after the mutation. By predicting the binding pockets of the WT protein and three mutant proteins (L71A, Y106A, and L114A), we observed the binding pockets of these three mutant proteins changed in shape, size, surface area and depth to different degrees. Compared to the WT protein, the volume of the binding pockets in all three mutant proteins was reduced, with L114A showing the most significant reduction, almost a quarter less than the WT protein. This reduction in volume may affect the binding of ligands to the protein, as the binding of a ligand to a protein is highly specific, similar to a key fitting into a lock. Successful binding requires the ligand’s shape and chemical properties to match those of the protein’s binding site (Li et al., 2015; Du et al., 2016). Li et al. (2015) demonstrated that when the ligand’s volume is too large to fit the binding pocket, more collisions between the ligand atoms and pocket can occur upon entry into the binding pocket (Li et al., 2015). Therefore, we speculate that a significant decrease in the volume and depth of the binding pocket may hinder the ligand’s ability to enter the pocket effectively, resulting in weakened binding ability. This could be the primary reason for the reduced binding ability of the L114A mutant protein to ligands.

## Data Availability

The datasets presented in this study can be found in online repositories. The names of the repository/repositories and accession number(s) can be found below: https://www.ncbi.nlm.nih.gov/genbank/, OR499868.
