# Supplementary material for: Ligand-binding properties of XaffOBP9, a Minus-C odorant-binding protein from Xyleborus affinis (Coleoptera: Curculionidae: Scolytinae)
Source: Front Physiol. 2024 Jan 3;14:1326099. doi: 10.3389/fphys.2023.1326099 (PMC10791897; doi:10.3389/fphys.2023.1326099)

**Supplementary Material**

**Table S1** Primers used in RT-qPCR, vector construction and site-directed.

| Purpose | Name | Fordward primer (5’→3’) | Reverse primer (5’→3’) |
| --- | --- | --- | --- |
| RT-qPCR | XaffOBP1 | TATTGCTGTTTCGGCTA | TCTTCATTGATTGTGGC |
|  | XaffOBP2 | TCTTATTGCTGCCGCTT | TCCGTCTTCGTTCTGCC |
|  | XaffOBP3 | ATAGTTCATTCGCTGTGC | TAAGGGCTGTTTCGTCT |
|  | XaffOBP4 | GACAAACAAAAGCAACTCC | TTGGCATCAATCACCTG |
|  | XaffOBP5 | GGAATACCGTCCATCAA | AACAATCTCATCCACTTTAG |
|  | XaffOBP6 | CACCATAAAGACAAGCACCAC | AGCATCGGCAGAACCTC |
|  | XaffOBP7 | TTTTGGCAGTGTTGTTG | CTACCATCGCATCTACG |
|  | XaffOBP8 | TTCGCCACAGTTTTCGGT | AGCCTTCTGCCATTTCAA |
|  | XaffOBP9 | GCTGTGGCTTTAGTCGGG | GATTGGCTGTGTCCTGGT |
|  | XaffOBP10 | GACAAACAAAAGCAACTCC | TTGGCATCAATCACCTG |
|  | β-Actin | ATGTGTGACGAAGAAGTTG | GTCCCAGTTGGTTACGA |
| Vector construction | pET-28a/XaffOBP9 | CATGCCATGGACTTAACCGATGAACAGAAG | ATATGCGGCCGCAAATACAGTTAAATGAGT |
| Site-directed | L71A | GCTGATGTGGTCAAGCAGAAAGCAGGGGGAGCCC | GGGCTCCCCCTGCTTTCTGCTTGACCACATCAGC |
|  | Y106A | CTTTCCAAAGTTTCAAGTGTGCCTAGAGAGCACCCCCTACTC | GAGTAGGGTGCTCTCATAGGCACACTTGAAACTTTGGAAAG |
|  | L114A | ACTATGAGAGCACCCCTACTCATGCAACTGTATTTGCGGCC | GGCCGCAAATACAGTTGCATGAGTAGGGGTGCTCTCATAGT |

Note: the restriction site is indicated by underlining.

**Table S2** Transcriptome assembly results of *Xyleborus affinis*

| Length Range | Transcript | Unigene |
| --- | --- | --- |
| Total Number | 67,228 | 27,786 |
| Total Length | 180,448,847 | 34,720,674 |
| N50 Length | 4,781 | 2,761 |
| Mean Length | 2684.13 | 1249.57 |

**Figure legend**

**Figure S1.** The cDNA and deduced amino acid sequences of XaffOBP9. Underlines indicate the predicted signal peptides. The conserved Cys sites are indicated in black boxes. The asterisk indicates the translation–termination codon.

**Figure S2.** Alignment of XaffOBP9 with OBP genes from other coleopteran insects. *Dendroctonus ponderosae* (Dpon), *Pagiophloeus tsushimanus* (Ptsu), *Dendroctonus armandi* (Darm), *Pyrrhalta maculicollis* (Pmac).

**Figure S3.** The 3D structure of XaffOBP9 constructs using I-TASSER. Nt, N-terminal; Ct, C-terminal; ɑ, ɑ-helix.

**Figure S4.** Evaluation of 3D model structure quality. (A) Error values of XaffOBP9 model structure residues evaluated by using ERRAT. (B) Ramachandran plots of model of XaffOBP9. Red, most favored regions, including A, B and L areas; Yellow, additional allowed regions, including a, b, l, p areas; Pale yellow, generously allowed regions, including ~a, ~b, ~l, ~p areas; White, disallowed regions. (C) Verify-3D scores for XaffOBP9 model. a, Amino acid; A, Alanine; C, Cysteine; D, Aspartic acid; E, Glutamate; F, Phenylalanine; G, Glycine; H, Histidine; K, Lysine; M, Methionine; N, Asparagine; Q, Glutamine; S, Serine; T, Threonine; V, Valine; Y, Tyrosine.

**Figure S5.** Expression and purification of three mutants. (A) SDS–PAGE analysis of the crude extracts of three mutants from the bacterial pellets before (−) and after (+) induction with IPTG. Lane M, molecular weight markers of standard protein; Lane 1-2, supernatant and sediment of induced BL21 bacteria within pET-28a/L71A vector; Lane 3-4, supernatant and sediment of induced BL21 bacteria within pET-28a/Y106A vector; Lane 5-6, supernatant and sediment of induced BL21 bacteria within pET-28a/L114A vector. (B) SDS-PAGE analysis of the recombinant of three mutant proteins expressed in *E. coil* BL21(DE3) and purified using Ni-NTA 6FF. Lane M, molecular weight markers of standard protein; Lane 1-2, sediment of induced BL21 bacteria within pET-28a/L71A vector and purified sample; Lane 3-4, sediment of induced BL21 bacteria within pET-28a/Y106A vector and purified sample; Lane 5-6, sediment of induced BL21 bacteria within pET-28a/L114A vector and purified sample.

**Figure S1.**

**

**

**Figure S2.**

**
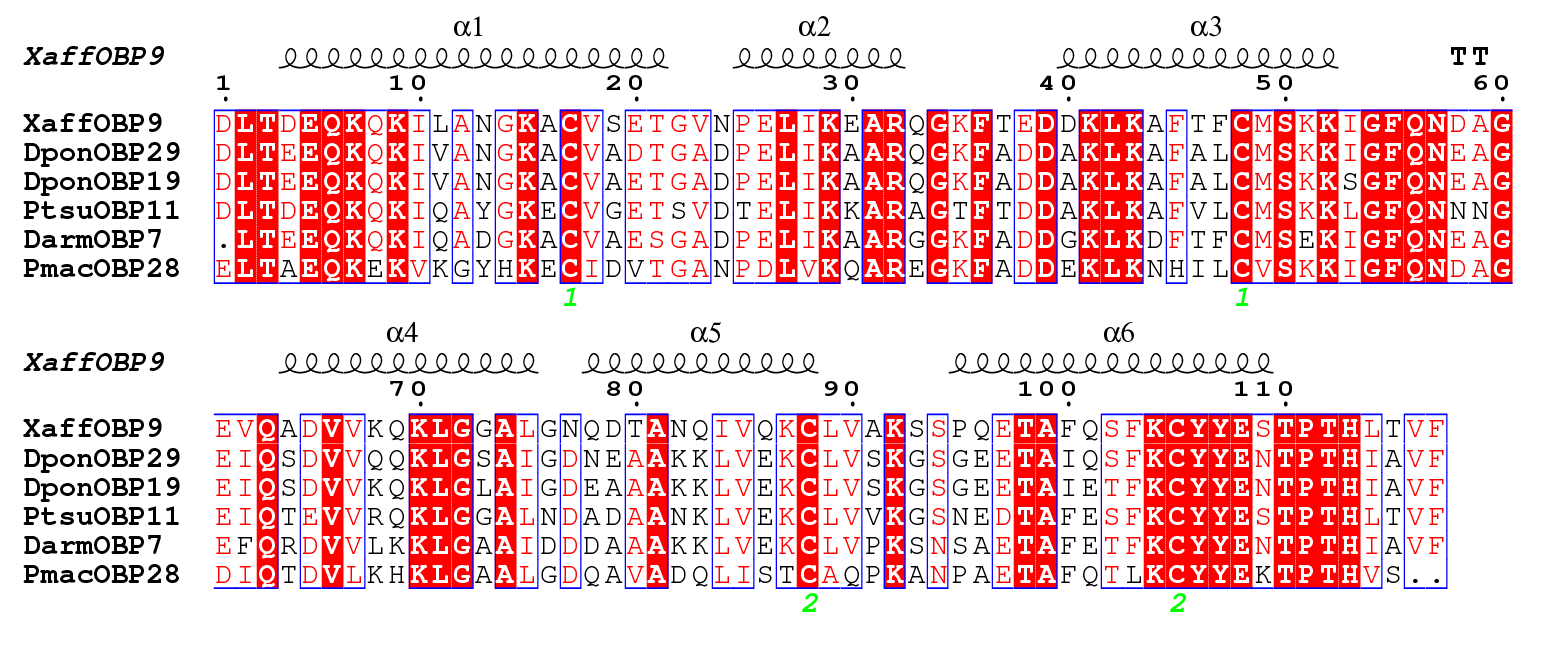
**

**Figure S3**


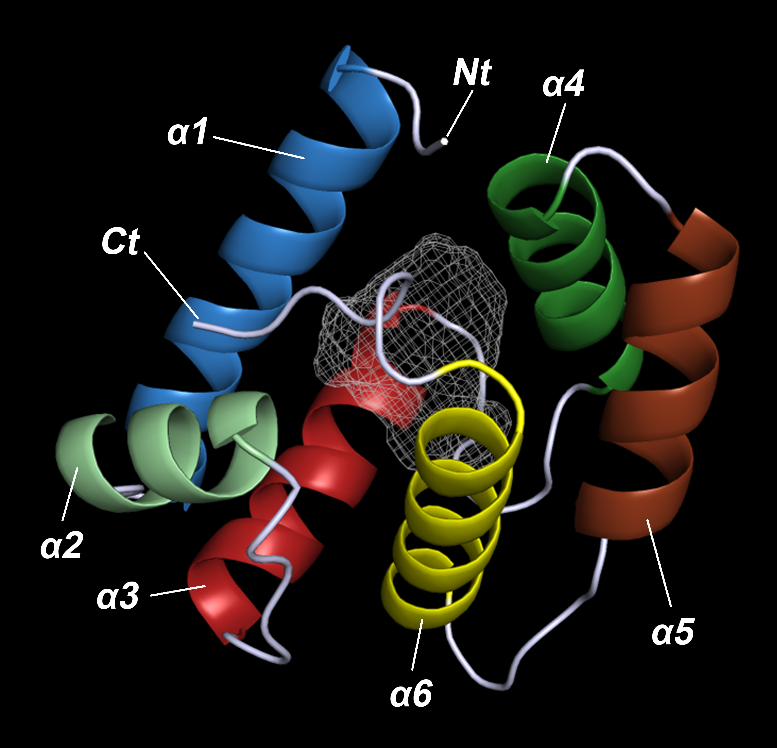


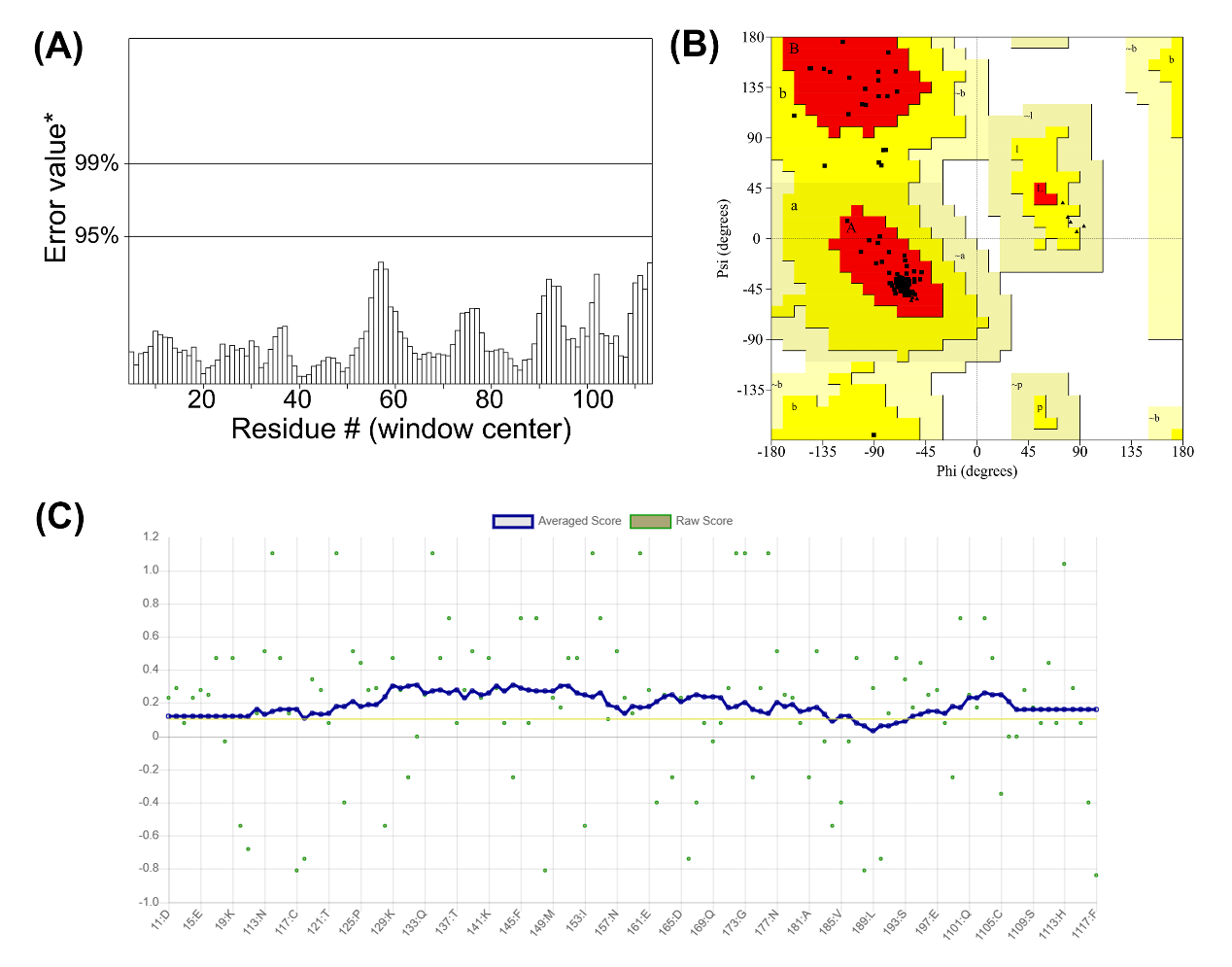
**Figure S4**

**Figure S5**


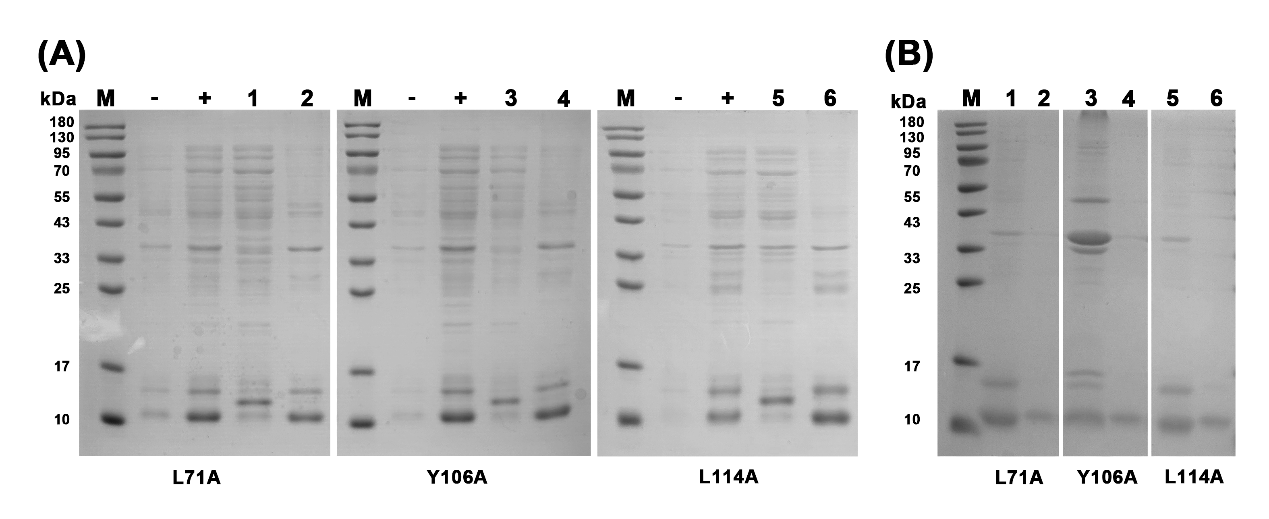

Supplement: Supplementary file 1 [file Table1.DOCX]
